# Evaluation of State Comprehensive Cancer Control Plans for Genomics Content

**DOI:** 10.5888/pcd9.120190

**Published:** 2012-12-20

**Authors:** Jason D. Laufman, Debra Duquette, Angela Trepanier

**Affiliations:** Author Affiliations: Debra Duquette, Michigan Department of Community Health, Lansing, Michigan; Angela Trepanier, Wayne State University, Detroit, Michigan.

## Abstract

**Introduction:**

Comprehensive Cancer Control (CCC) plans address cancer burden at the state level through consolidation of activities and collaboration among stakeholders. Public health genomics strategies are increasingly important in prevention and treatment of cancer. The objectives of this study were to assess the extent to which CCC plans have incorporated genomics-related terms since 2005, determine which of the 3 core public health functions were fulfilled by genomics components, and identify facilitators of and barriers to integration of genomics.

**Methods:**

We reviewed 50 CCC plans in 2010 to assess use of 22 genomics-related terms. Among plans that used the term *genetics* or *genomics*, we examined the plan for inclusion of genomics-related goals, objectives, or strategies and documented the 3 core public health functions (assessment, policy development, and assurance) fulfilled by them. We surveyed plan coordinators about factors affecting incorporation of genomic strategies into plans.

**Results:**

Forty-seven of 50 (94%) plans included at least 1 genomics-related term. Thirty-two of 50 (64%) plans included at least 1 genomics-related goal, objective, or strategy, most encompassing the core function of assurance; 6 state plans encompassed all 3 core functions. Plan coordinators indicated that genomics is a low priority in state public health; barriers to incorporation included lack of sufficient staff and funding.

**Conclusion:**

Incorporation of genomic terms into state CCC plans increased from 60% in 2005 to 94% in 2010, but according to plan coordinators, genomics has not grown as a priority. Identification of partnerships and resources may help increase the priority, encourage incorporation, and guide the eventual success of public health genomics in state plans. Strong partnerships with state public health departments, health care providers, and the research community are useful for integration.

## Introduction

Comprehensive Cancer Control (CCC) is a collaborative community-based approach that uses an “integrated and coordinated approach to reduce cancer incidence, morbidity, and mortality” (1). Genomics aids in cancer prevention, detection, and treatment through early recognition, intervention, and efficient treatment (2–4). Genomics refers to the study of the genome as a whole, including single genes and the interaction of multiple loci and the environment. Single-gene genomic applications, such as cascade testing for Lynch syndrome, have a potential effect in cancer prevention in high-risk families (5). The fundamental tool of genomics — collecting family history — is a low-cost and effective method to identify people at increased risk and to promote evidence-based screening (5).

The 3 core functions of public health are assessment, the collection and dissemination of data; policy development, the creation of standards; and assurance, ensuring quality and accessibility of services (6). A survey of public health and laboratory officials in 2001 identified that assurance was the most common function of public health genomics (82%); however, the investigators predicted more balance among the 3 functions (7).

One other review of genomic content of state CCC plans in 2005 found that 18 (60%) of the 30 existing plans contained genomics content, as determined by searching the plans for genomic terms; however, the way in which terms were used varied (8). Today, all 50 states have a CCC plan. Genomics recommendations from the US Preventive Services Task Force (USPSTF), Evaluation of Genomic Applications in Practice and Prevention (EGAPP), and Healthy People 2020 have been released, and the Genetic Information Nondiscrimination Act (GINA) has been enacted (9–12). The effect of these developments on CCC plans is unknown.

The objectives of this study were to assess the extent to which CCC plans have incorporated genomics-related terms since 2005, determine which of the 3 core public health functions were fulfilled by the genomics components, and identify facilitators and barriers to integration of genomics.

## Methods

We conducted a qualitative analysis of state CCC plans to evaluate genomics content and surveyed state CCC plan coordinators to ascertain factors that affect incorporation of genomics strategies into plans.

### Qualitative analysis of state CCC plans

The Centers for Disease Control and Prevention, National Comprehensive Cancer Control Program (NCCCP) maintains a database of all CCC plans (13). All 50 state CCC plans identified on the NCCCP website as of August 15, 2010, were analyzed for genomics content. We made no attempt to find alternative or updated versions. We excluded tribal government or territorial plans from the analysis to maintain consistency and allow comparison to the findings from Irwin et al (8). Five plans did not indicate the year in which the plan was published or scheduled to take effect; the start year on the remaining 45 plans ranged from 2001 to 2010. Fourteen plans did not specify a completion date, but 27 of the remaining 37 plans specified a completion date on or before the year 2010 (range 2005–2015). We included the expired plans because they were the plans of record in the NCCCP database. The Irwin study (8) did not identify the plans reviewed, so we could not assess overlap between the 2 studies.

We searched each CCC plan by using an automated text search function (Box). We used 7 search terms used in the Irwin study (8) and 15 search terms identified by the study team. Using methods described by Irwin et al (8), we created a narrative assessment of each plan; it described the context and depth of the genomics components. We tabulated use of the following 10 terms because they were common and allowed for quantitative comparison between plans: *gene*, *genetics*, or *genomics*; *family history*; *heritability*, *hereditary*, or *heredity*; BRCA, and *Lynch syndrome* or *HNPCC* (hereditary nonpolyposis colorectal cancer). Summaries of the context of use were recorded by the principal investigator (J.L.) and reviewed by another investigator (D.D.). Additionally, some states included discrete and actionable genomics-related goals, objectives, or strategies. If a genomics-related goal, objective, or strategy was identified, the goal, objective, or strategy was categorized as filling 1, 2, or all 3 of the core functions of public health (assessment, policy development, and assurance) as defined by the Association of State and Territorial Health Officials (6). Each of these items was recorded verbatim and analyzed independently by the investigators (J.L., D.D.), and inconsistencies were discussed until a consensus was reached. Our analysis did not order these categories hierarchically to maintain consistency between plans.

**Box. Genetics- and Genomics-Related Terms Used To Search State Comprehensive Cancer Control Plans**

**Table:** 

**Search Terms Used By Irwin et al (8)**	**New Search Terms**
Genetics	Genetic counselor
Genomics	Heredity
Gene	Hereditary
Family history	Genetic services
DNA	Relative
First-degree relative	Risk assessment
Heritability	High Risk
	Genetic test
	Genetic testing
	BRCA
	HNPCC
	Lynch syndrome
	Gene expression profiling
	Cancer genetic experts
	Geneticist

### Survey of state CCC plan coordinators

We created a semiquantitative survey using the survey developed by Irwin et al (8) as a guide. Open-ended responses from the Irwin survey (eg, barriers, successes) were converted to multiple-choice questions with the option of open responses (Appendix). The survey also included questions about planning and writing of the CCC plan, implementation of genomics objectives, and characterization of the role and priority of genomics in CCC. The survey was made available online via Survey Monkey (http://www.surveymonkey.com/) or in a paper format. A representative from each of the states whose plan contained at least 1 term related to genetics or genomics was invited to participate in the survey. We collected responses during April and May 2011. The survey portion of this study was approved by the Wayne State University institutional review board.

We visited each state CCC website to obtain e-mails, addresses, and telephone numbers for state CCC plan representatives. If an e-mail was available, we sent an invitation to participate in the survey, including a link to the survey. If no e-mail was available, we mailed a packet that included a copy of the survey and instructions for accessing the survey online. People who had not yet completed the survey or declined to participate were contacted via telephone 2 weeks after the initial invitation. If no telephone contact could be made, an e-mail or second mailing was sent to the address used previously. 

We used descriptive statistics to analyze data. Percentages and responses are presented as proportions of states that answered each question. 

## Results

### Review of state CCC plans

Of the 50 state CCC plans reviewed, 47 (94%) contained at least 1 genomics-related term. Of these, most mentioned *family history* (43 of 47, 91%) and *gene* or *genetics* (40 of 47, 85%). Fewer plans mentioned *genomics* (11 of 47, 23%) or specific genetic conditions such as *hereditary breast ovarian cancer* or BRCA (18 of 47, 38%) or *Lynch syndrome* or *HNPCC* (6 of 47, 13%).

Overall, 32 of 47 states (68%) that mentioned genetics or genomics had a genomics-related objective, goal, or strategy (Table). These objectives, goals, and strategies had similar themes, including educating the public or health care providers about the importance of genetics and/or family history, collaborating with academic institutions, researchers, or workgroups, identifying people at risk for a genetic susceptibility to cancer, and supporting research. The most common goal was to increase access to genetic risk assessment services such as genetic counseling or genetic testing, including reimbursement for genetic risk assessment services (24 of 32, 75%). Family history was the second most common theme (18 of 32, 56%), including education of the public or health care providers or development of a family history tool. Other goals included preventing genetic discrimination (5 of 32, 16%) and encouraging research in the field of genetics and genomics (5 of 32, 16%).

**Table:** Comprehensive Cancer Control (CCC) Plans,^a^ by State, Date Range, and Genetic/Genomic Goal, Objective, or Strategy Genetics or Genomics Component

State	Period Covered by CCC Plan		Genomics-Related Goal, Objective, or Strategy	Core Public Health Functions^b^ (6)		
	
	Began	Ends		Assessment	Policy	Assurance
Alabama	2006	2010	No			
Alaska	2005	2010	Yes			X
Arizona	NS	NS	No			
Arkansas	NS	NS	Yes			X
California	2004	NS	No			
Colorado	2005	2010	Yes			X
Connecticut	2005	2008	Yes			X
Delaware	NS	NS	No			
Florida	2003	2006	No			
Georgia	2008	2012	No			
Hawaii	2004	2009	Yes			X
Idaho	2006	2010	No			
Illinois	2005	2010	Yes	X		X
Indiana	2005	2008	No			
Iowa	2006	2011	Yes		X	X
Kansas	2005	NS	No			
Kentucky	NS	NS	No			
Louisiana	2004	2009	No			
Maine	2006	2010	Yes			X
Maryland	2004	2008	Yes	X		X
Massachusetts	2006	2011	Yes		X	X
Michigan	2009	2015	Yes	X	X	X
Minnesota	2005	2010	Yes	X	X	X
Mississippi	2006	2011	Yes	X	X	X
Missouri	2004	NS	No			
Montana	2006	2011	Yes			X
Nebraska	2004	2010	Yes			X
Nevada	NS	NS	No			
New Hampshire	2005	2010	Yes		X	X
New Jersey	2008	2012	Yes			X
New Mexico	2007	2011	Yes	X	X	X
New York	2003	2010	Yes	X		X
North Carolina	2010	NS	Yes		X	X
North Dakota	2006	2010	Yes			X
Ohio	2006	2010	Yes			X
Oklahoma	2007	NS	No			
Oregon	2005	2010	Yes	X	X	X
Pennsylvania	2003	NS	Yes	X		
Rhode Island	2007	NS	No			
South Carolina	2005	2010	Yes	X		X
South Dakota	2005	2010	Yes		X	
Tennessee	2009	2012	Yes			X
Texas	2005	NS	Yes			X
Utah	2006	2011	Yes		X	X
Vermont	2006	2010	Yes			X
Virginia	2001	2005	No			
Washington	2004	2008	Yes	X	X	X
West Virginia	2007	NS	No			
Wisconsin	2005	2010	Yes			X
Wyoming	2006	2010	No			

Abbreviation: NS, not specified.

a Plans obtained in August 2010 (12).

b Core functions categorize the nature of the genetic/genomic goal as assessment, assurance, or policy development based on definitions set forth by the Association for State and Territorial Health Officials (6). All states except Kentucky, Oklahoma, and West Virginia included at least 1 core function.

Overall, 30 of the 32 (94%) states’ goals, objectives, or strategies fulfilled the function of assurance; 11 of the 32 (34%), assessment; and 12 of the 32 (37%), policy development (Table). Six states had objectives or strategies that encompassed all 3 core public health functions, including 3 of the 4 states that received a 5-year cooperative agreement with CDC from 2003 through 2008 to incorporate genome-based knowledge into disease prevention (Michigan, Minnesota, and Oregon). All states except Kentucky, Oklahoma, and West Virginia included at least 1 core function. Examples of the strategies related to assessment included assessing the effect of direct-to-consumer marketing of genetic test campaigns and review of health plan policies for consistency with USPSTF guidelines for *BRCA* counseling. Examples of the strategies related to policy development included advocating for third-party payment of genetic counseling, testing for the uninsured and underinsured, and advocating for genetic counseling state licensure. Examples of strategies related to assurance included provider and public education about genomics.

Plans that mentioned genomics but did not mention an objective, goal, or strategy often mentioned genomics in terms of genetic risk, health disparities, or health insurance discrimination. Many plans provided extensive information on the state’s cancer burden or cancer risk factors that included an explanation of genomics concepts. Other plans described family history as a risk factor for certain cancers and detailed the genetics of hereditary breast and ovarian cancer and Lynch syndrome.

### Survey

Thirty-five of the 47 plans that included genomics-related terms were contacted via e-mail from published e-mails or e-mails provided on initial contact by telephone, and the remaining 12 surveys were mailed to the state contact address published on the CDC NCCCP website (13). Twenty surveys were completed online, and 2 surveys were submitted through mail. Three states that completed the online survey provided only demographic information and were excluded from the analysis (overall response rate, 40% [19 of 47]). Fifteen respondents indicated that they were the states’ CCC plan manager or coordinator, 1 respondent was a designated contact person for cancer genomics, 1 respondent was a regional chairperson, and 1 respondent did not identify a position.

Seventeen of 19 respondents identified sectors involved in the planning and writing of the CCC plan, including hospitals (17 of 17), support groups or advocacy organizations (17 of 17), local public health organizations (16 of 17), and academia (15 of 17). These 4 sectors were also identified as being the biggest champions of genomics integration into the CCC plan. Eleven respondents identified key facilitators for the integration of genomics concepts, including partnership with the health care community and research community (7 of 11) and the state department of health (5 of 11). In an open-response question asking states to identify the organization or individual who provided the greatest amount of support to integration of these concepts, 4 respondents identified genetic counselors.

When asked, most respondents (10 of 18) believed that awareness of genomics increased between the years of 2000 and 2006. However, many different events or circumstances were noted as the cause of this increase in awareness, including the human genome project, the identification of *BRCA1/2*, CDC genomics funding, the promise of gene therapy, and collaboration with members of the public health community, health care providers, and researchers. No states identified EGAPP, USPSTF recommendations, GINA, or Healthy People 2020 objectives. Eleven of 15 respondents indicated that genomics is not a priority or somewhat not a priority, while no states rated genomics as a high priority. Of the 6 respondents from states that could assess the change in priority of genomics, 3 felt the priority had increased in the last 5 years, and 3 felt that the priority had decreased.

When asked, only 3 respondents were able to identify barriers to the implementation of genomic concepts; these barriers included genomics being a low priority (2 of 3), time constraints (2 of 3), lack of sufficient staff or leadership (2 of 3), and lack of funding (3 of 3). The low response rate to this question may be due to the fact that only 4 respondents indicated they had implemented genomic components within the CCC plan; 3 respondents reported having accomplished their objectives. An open-response question asking respondents to identify partnerships and resources key to implementing genomic components was answered by 10 respondents; 4 identified increased funding and 3 identified stronger partnerships with health insurance companies and managed care agencies. Other respondents identified stronger partnerships with researchers, health care workers, and academia; development of evidence-based strategies at the national level; and examples of successful CCC plans that implement genomics concepts.

## Discussion

Our findings on the use of genomics concepts in CCC plans are similar to those in the Irwin study: many plans focused on education, collaboration, and family history and risk assessment (8). Although more plans in our study used genomics terms than plans in the Irwin study, the scope and depth of coverage was inconsistent.

Interestingly, many plans that have goals, strategies, or objectives related to genetics or genomics focus on the core function of assurance (30 of 32) and less on assessment (11 of 32) and policy development (12 of 32). These core functions have been described as cyclical: assessment leads to policy development, and policy development results in assurance (6). However, the genomics-related objectives identified in our study appeared to function in reverse: for example, a family history tool was developed before its need was assessed. Such a lack of assessment hinders the ability to measure the effect of genomics interventions.

Another approach to translating genomic discoveries into population health has been described as a continuum of 4 linear phases (14). Phase 1 translates a basic genome-based discovery into a health application; Phase 2 creates evidence-based guidelines; Phase 3 moves the evidence-based guidelines into practice; Phase 4 evaluates the effect on population health. Three percent or less of genomics research occurs in Phase 2 through 4 (14). Some have questioned the large amount of spending on recent genomic research and the lack of genomics-related benefits in medicine (15). These questions may be influencing the decreased prioritization of genomics reported by several survey respondents. 

Although only 3 respondents reported on barriers, the barriers were similar to those reported by Irwin et al (8) and by others examining the incorporation of evidence-based initiatives into state CCC plans; they may not be unique to genomics (16,17).

The strengths of this study include the finite, well-defined sample. Having access to all 50 state CCC plans through the CDC’s NCCCP website allowed for comprehensive analysis. The availability of contact information allowed for invitation and representation of all states that chose to participate. The semiquantitative survey method permitted objective analysis while encouraging respondents to share experiences leading to the identification of barriers and successes, which may guide future studies and CCC planning. Additionally, the survey allowed plan coordinators an opportunity to comment on their state’s upcoming revisions if a plan was about to or had expired.

This study has limitations. One limitation is that 27 out of 32 plans with a published end date were set to expire on or before 2010. Once plans are updated, a reanalysis should be performed, particularly because of recent genomics recommendations and legislation. Another limitation is that we could not assess overlap between plans used for our analysis and analysis by Irwin et al (8). Nonetheless, this study reflects an analysis of the current plans identified on the NCCCP website (13). Survey results were potentially biased because of the use of a mail and e-mail format; only 2 mail-based surveys were returned. States that do not publish an e-mail address may not have a CCC coordinator, and results may represent only a subset of states. The response rate of 40% is low, especially compared with the response rate of 89% for the Irwin study (8). Finally, some respondents decided not to answer a wide range of questions; this lack of response limited our data set.

Overall, we affirmed our hypothesis that more states are incorporating genomics into state CCC plans. However, the scope and depth of coverage is narrow, with many plans focusing on assurance. How the incorporation of genomics-related concepts affects cancer control is unclear, but assessment will be crucial measuring the impact as it may take years to recognize the effects. USPSTF and EGAPP recommendations and Healthy People 2020 genomics-related objectives should serve as a baseline for integrating genomics into state CCC plans. Other chronic disease plans (eg, asthma, diabetes, cardiovascular disease, dementia) are being created and can also benefit from the research on the successes and barriers of genomics integration into CCC plans. Finally, learning from the successes and barriers of states may lead to standardization of language and goals for future plans, including a cohesive national CCC plan.

## Appendix

What state do you represent?

1. Are you the Comprehensive Cancer Control (CCC) Plan manager? If no, please specify your position.

Yes

No

Other (specify):

1a. How many years have you been the CCC Plan manager?

0–1 years

1–2 years

2–3 years

3–4 years

5+ years

2. How many years have you worked for the CCC section of your health department?

0–1 years

1–2 years

2–3 years

3–4 years

5+ years

3. What is your educational background?

High school diploma/GED

Associate degree

Bachelor degree

Master degree

Other postgraduate degree (eg, JD, MD) (Please specify):

4. Which of the following sectors were involved in the planning/writing of the CCC plan (check all that apply):

Academia

Hospitals/health care providers

Private sector/industry

Support groups/advocacy organizations

Faith-based groups

Local public health

Media

Unknown/don’t know

Other (Specify)

4a. Of these groups (academia, hospitals/health care providers, private sector/industry, support groups/advocacy organizations, faith-based groups, local public health, media), who do you perceive as the biggest champion of genetics/genomics integration (please rank from lowest to highest)?  5. When was genetics and/or genomics identified as relevant in public health and cancer prevention and control?

1990–1995

1995–2000

2000–2002

2002–2004

2005–2006

2007–2008

2009

2010

Don’t know/Unknown

5a. How was genetics/genomics identified as a priority in public health and cancer prevention/control (such as an event or circumstance)?

6. Please identify all of the facilitators that were key to integration of the genetics/genomics components of the CCC plan

Strong partnerships within the state department of health

Strong partnerships with the health care provider community

Strong partnerships with the research community

High priority of genetics/genomics within state public health department

Genetic counselor on staff

Additional funding sources

Provision of continuing education credits for professions

Unknown/don’t know

Other (please specify):

6a. What organization or individual was your greatest source of support to implementation of genomics concepts?

7. Please describe the general process used to write the genetic/genomics section(s).

8. Did you experience any barriers to the implementation of specific genetic/genomic components?

Yes

No

Don’t know/Unknown

8a. What barriers have you encountered?

Lack of a strong partnership with the research/medical community

Lack of support within the department

Low priority

Lack of sufficient staff/leadership

Lack of sufficient funding

Time constraints

Misperceptions or misinformation about genetics/genomics

Lack of evidence base

Lack of demonstrable outcomes

Other (please specify):

8b. How have you overcome these barriers?

9. To date, have you had the opportunity to implement the genetic/genomic components within your plan?

Yes

No

Don’t know/Unknown

9a. Have you accomplished your assigned goal(s)/objective(s)?

Yes

No

Don’t know/Unknown

9b. If yes, what was your greatest accomplishment to date? If possible, please include an explanation of the process outcomes and population impact.

9c. If no, why do you feel you have not accomplished your assigned goal/objective?

10. What is the current priority level of genomics in your state health department?

High priority

Somewhat a priority

Somewhat not a priority

Not a priority

Don’t know/Unknown

10a. How has the priority changed over the last 5 years?

Increased

Stayed the same

Decreased

Cannot assess

11. Have you begun the process of drafting a subsequent CCC?

Yes

No

11a. If yes, have the genetics/genomics components changed?

Yes

No

11b. Are there plans to incorporate information on specific genetic cancer syndromes (eg, Hereditary Breast and Ovarian Cancer Syndrome, Lynch syndrome)?

Yes

No

Incorporated into previous plan

11c. Which syndromes are being considered for incorporation in your future plan?

Hereditary Breast and Ovarian Cancer Syndrome (BRCA1/2)

Lynch syndrome

Other (specify):

12. What types of partnerships and resources would be helpful in further implementing the genomics components of your state plan?

13. What public health genomics training resources have you utilized?
